# Supplementary material for: Collaborative intelligence in AI: Evaluating the performance of a council of AIs on the USMLE
Source: PLOS Digit Health. 2025 Oct 9;4(10):e0000787. doi: 10.1371/journal.pdig.0000787 (PMC12510544; doi:10.1371/journal.pdig.0000787)
Supplement: S2 File — (ZIP) [file pdig.0000787.s002.zip › S2 Data Raw data files containing unprocessed question inputs and LLM outputs including transcripts of deliberations/STEP3_Question-131-1_Type-MC-NJ.docx]

**Facilitator** **[Round 1]:** A 32-year-old woman comes to the emergency department because of a 3-day history of worsening fever, dry cough, and shortness of breath. She also has had abdominal discomfort, diarrhea, and nausea, but she has not vomited. She returned home from a business trip to a midwestern city 2 days ago. She has had no known contact with any ill individuals. She had been generally healthy and medical history is unremarkable. She takes no medications. Her last menstrual period was 6 weeks ago and she thinks she might be pregnant. She does not smoke cigarettes, drink alcoholic beverages, or use illicit drugs. She is married and uses no contraception. She appears well developed and well nourished but is short of breath and lethargic. Vital signs are temperature 38.3°C (101.0°F), pulse 104/min, respirations 28/min, and blood pressure 100/60 mm Hg. Pulse oximetry on room air shows an oxygen saturation of 92%. Auscultation of the chest discloses crackles at the right lung base, tachycardia, a normal S1 and S2, and no other abnormalities. Abdominal examination discloses no abnormalities. Petechiae are present over both lower extremities. Results of laboratory studies are shown: Serum ALT - 80 U/L AST - 63 U/L Urea nitrogen - 14 mg/dL Creatinine - 1.7 mg/dL Na⁺ - 130 mEq/L K⁺ - 3.9 mEq/L Cl⁻ - 104 mEq/L HCO3⁻ - 16 mEq/L Iron, total - 18 μg/dL Iron binding capacity, total - 428 μg/dL (N=250–350) Iron saturation - 5% (N=20–50) β-hCG - Positive Blood Hematocrit - 30.3% Hemoglobin - 10.2 g/dL WBC - 11,300/mm³ MCV - 76 μm³ Platelet count - 104,000/mm³ Red cell distribution width - 16% (N=11.5–14.5) Which of the following is the most appropriate intravenous pharmacotherapy at this time? (A) Cefuroxime alone (B) Cefuroxime and azithromycin (C) Levofloxacin alone (D) Levofloxacin and ticarcillin (E) Piperacillin-tazobactam

**AI_A:** RESTATING THE QUESTION: The clinical scenario presents a 32-year-old woman in potential early pregnancy who presents with fever, respiratory and gastrointestinal symptoms, along with petechiae. The laboratory results suggest possible infections, anemia and thrombocytopenia. The question is to select the most suitable intravenous treatment for this patient's condition. The options consist of various antibiotics, indicative of a possible infection. PLANNING: The woman's symptoms of fever, cough, and shortness of breath together with her recent travel to a midwestern city hint at a respiratory infection, potentially community-acquired pneumonia (CAP). The gastrointestinal symptoms could indicate a systemic infection. However, the presence of petechiae, an elevated total iron binding capacity, low iron saturation, combined with possible anemia and thrombocytopenia in blood tests, might suggest a blood disorder or disseminated intravascular coagulation (DIC). The increased ALT and AST levels also point to possible liver dysfunction. Given her positive β-hCG test and absence of contraception with recent sexual activity, she is likely pregnant. This complicates the choice of an intravenous medication, as we must consider the potential impact on the fetus. CLINICAL REASONING: These clinical indications of community-acquired pneumonia (CAP) can be addressed by several antibiotics, including cefuroxime, azithromycin, levofloxacin, ticarcillin, and piperacillin-tazobactam. However, fluoroquinolones like levofloxacin are generally avoided in pregnancy due to potential fetal risks. Therefore we should consider a combination of macrolide (azithromycin) and a cephalosporin (cefuroxime), which are generally safe in pregnancy. Macrolides cover atypical organisms and cephalosporins cover typical bacterial causes of pneumonia. CONCLUDING: Despite the potential blood disorder and liver dysfunction, they require further confirmation and the immediate need is to address the possible pneumonia due to the urgent nature of her respiratory symptoms. Given her pregnancy, the very clear respiratory symptoms, and potential for harm to the fetus from certain antibiotics, a combination of cefuroxime and azithromycin is indicated for CAP. RESPONSE: Therefore, the most appropriate intravenous pharmacotherapy at this time for this patient is (B) Cefuroxime and azithromycin.

**AI_B:** RESTATING THE QUESTION: The query concerns a woman, aged 32, who presents with symptoms of fever, dry cough, breathlessness, abdominal discomfort, diarrhea, and lethargy. She recently returned from a trip to a midwestern city and is potentially pregnant. Physical examination and laboratory findings reveal signs of pneumonia, septicemia and iron deficiency anemia. The question is asking what would be the best choice of intravenous medication to administer at this point in time, with the options being various antibiotics -(A) Cefuroxime alone, (B) Cefuroxime and azithromycin, (C) Levofloxacin alone, (D) Levofloxacin and ticarcillin, (E) Piperacillin-tazobactam. PLANNING: To answer this query, we should consider a number of assumptions: Is the condition bacterial or viral? Could the woman's potential pregnancy be causing or exacerbating her symptoms? Is there any other underlying condition? If we hypothesize that the symptoms are due to a bacterial infection (suggested by her recent trip, fever, and leukocytosis), the woman would likely benefit from antibiotics. Further, her possible pregnancy requires us to choose a safer option considering fetal wellbeing. CLINICAL REASONING: Her clinical presentation suggests the development of community-acquired pneumonia, evident by the presence of fever, dry cough, and shortness of breath along with characteristics lung auscultations. As per her blood investigation results, she also presents with signs of septicemia indicated by petechiae and leukocytosis. Additionally, she presents with iron deficiency anemia which is indicated by low iron and hematocrit levels, along with elevated red cell distribution width. The beta-hCG test also confirms her suspicion of being pregnant. Considering her clinical and laboratory findings and the aforementioned hypotheses, it's important to select an antibiotic regimen that is both effective against the causative pathogen usually involved in community-acquired pneumonia and safe in pregnancy. CONCLUDING: Given all these observations, it seems clear that the woman requires a tailored treatment plan that addresses her pneumonia, septicemia, and anemia in a way that is safe for a potential pregnancy. RESPONSE: Given the requirements just laid out, the most appropriate intravenous pharmacotherapy at this time would be (B) Cefuroxime and Azithromycin. Cefuroxime is a second-generation cephalosporin with broad activity against bacteria responsible for community-acquired pneumonia. Azithromycin, a macrolide, effectively covers atypical pathogens involved. Most importantly, both these antibiotics are safe to use in pregnancy. Please note, further management should also include treatment for her anemia and septicemia after identifying the underlying cause.

**AI_E:** RESTATING THE QUESTION: We are asked to decide which intravenous pharmacotherapy would be most suitable for a 32-year-old woman who has presented a variety of symptoms including fever, dry cough, shortness of breath, abdominal discomfort, diarrhea, and nausea. She demonstrates signs of potential infection such as increased respiration rate and temperature(Fever), lower oxygen saturation, evidence of crackles in the right lung base indicating potential pneumonia, and petechiae - small purplish spots on the skin caused by a minor bleed. Laboratory results show she also has a positive β-human chorionic gonadotropin (β-hCG) test indicating pregnancy and symptoms of malaise which is causing lethargy. Among the blood parameters are instances such as relatively lower platelet count, lower Iron saturation levels and higher total iron binding capacity which may indicate iron-deficiency anemia. PLANNING: Considering the patient's symptoms and clinical findings, the most likely assumption could be that she has acquired infection(pneumonia) and is also likely pregnant. The infection seems severe considering fever, raised white blood cells count, crackles heard at the lung base and decreased oxygen saturation. Furthermore, considering the mid-western travel history, and a possibility of contracting a zoonotic infection should also be assumed. The petechiae suggest likely septicemia or disseminated intravascular coagulation (DIC). Also, considering the patient's suspected pregnancy, teratogenecity of the potential drug should also be assessed. CLINICAL REASONING: From a clinical perspective, the respiratory and some constitutional symptoms such as fever, dry cough, and shortness of breath with the presence of crackles at the right lung base can be viewed as indicative of pneumonia. This, when correlated with recent travel history and being ill despite previously being generally healthy, can lead to suspicions of a zoonotic disease acquired from the Midwestern USA such as Histoplasmosis. The gastrointestinal symptoms displayed can also be associated with some respiratory infections. However, given her positive pregnancy test, the choice of any pharmaceutical therapy must consider its safety for the foetus. Macrolides like Azithromycin are generally considered safe during pregnancy. Lastly, the presence of petechiae cannot be overlooked as it may indicate septicemia or spreading infection. With these factors in consideration, a combined therapy highlighting a drug like Cefuroxime, a second-generation cephalosporin effective against community-acquired pneumonia, with Azithromycin may hold the highest therapeutic benefit. CONCLUDING: Due to the patient's symptoms including fever, dry cough, and shortness of breath, combined with the presence of crackles at the right lung base, a high probability of pneumonia is suggested. Given her Midwest travel history, a zoonotic infection must be considered. Moreover, with her positive pregnancy test, the chosen therapy must take teratogenicity into account. When considering all factors, a combined therapy of Cefuroxime and Azithromycin could offer the most beneficial support. RESPONSE: Therefore, the most appropriate intravenous pharmacotherapy at this time is (B) Cefuroxime and azithromycin.

**AI_C:** RESTATING THE QUESTION: The given scenario presents a 32-year-old female patient who has shown up at the ER with symptoms such as fever, dry cough, and shortness of breath of 3 days, plus abdominal discomfort, diarrhea, and nausea. The patient also returned just a few days ago from a trip to a midwestern region. She thinks she might be pregnant as she hasn't had a menstrual period for the past 6 weeks and she uses no contraception. Moreover, she demonstrates an oxygen saturation level of 92%, tachycardia, and crackles at the right lung base. Abnormally elevated ALT and AST, petechiae over lower extremities, positive β-hCG, and suboptimal hematology results raise suspicion about her health condition. The question asks to select the most fitting intravenous pharmacotherapy considering these patient symptomatology and lab results. PLANNING: The given symptoms might stem from different causes. Infection, such as pneumonia, given the ongoing fever, cough, and sounds from the lung base, are a concern. Another assumption is that the woman could have sepsis given the persistent fever, tachycardia, and respiratory distress - symptoms that correspond with the systemic inflammatory response syndrome (SIRS) criteria. The petechiae observed could indicate disseminated intravascular coagulation (DIC), which usually happens in the course of sepsis. Abnormal liver function tests and elevated total iron binding capacity could suggest potential liver issues. The anemia could be a result of some sort of infection, or it could be related to early pregnancy, as indicated by the positive β-hCG test. Considering the potential for pregnancy, it's critical to consider drugs that are safe in pregnancy. Defining the root cause and the most suitable drug are thus crucial. CLINICAL REASONING: With generalized symptoms of fever, cough, and shortness of breath along with the lung base crackles, we are led to believe she may have a lower respiratory tract infection. The fever, tachycardia, and petechiae on the lower extremities might suggest sepsis. Given her recent travels, she possibly could have contracted a disease from there. The blood test results confirm inflammation and possible sepsis (elevated WBC count, mild anemia, and thrombocytopenia). The impaired liver function tests (elevated AST and ALT) and augmented total iron binding capacity might be secondary to infection or liver issues due to sepsis. However, considering her current symptoms and the high likelihood of lower respiratory tract infection, it would be judicious to prioritize the treatment of potential pneumonia and sepsis. CONCLUDING: The patient presents signs consistent with a severe lower respiratory tract infection. With the fever, cough, and the observed lung sounds, an infection, such as pneumonia, is plausible, which could have developed into sepsis (with symptoms meeting 2 of the SIRS criteria). In a case like this, it would be crucial to initiate broad-spectrum antibiotic cover. RESPONSE: The antibiotic treatment should be broad spectrum considering the severity of symptoms and high suspicion of sepsis. As she is possibly pregnant, we must keep in mind the safety of drugs during pregnancy. Out of all listed antibiotic choices, (E) Piperacillin-tazobactam would be the most suitable one. It covers a broad range of organisms, including Pseudomonas aeruginosa, and is safe in pregnancy.

**AI_D:** RESTATING THE QUESTION: In other words, the woman presents with fever, respiratory and gastrointestinal symptoms, lethargy, and abnormal findings on physical and laboratory examination, including detected pregnancy. Her travel history to a midwestern city is a noted point of consideration. We are being asked to decide what medication should be given intravenously to treat her condition. DEFINING TERMS: 1. β-hCG - it is a hormone produced during pregnancy, and a positive β-hCG test suggests that the woman is likely pregnant. 2. ALT and AST - these are liver enzymes, and increased levels suggest a liver problem. 3. Creatinine - a waste product filtered by kidneys; increased levels can suggest kidney problems. 4. Hematocrit and Hemoglobin - these measurements are used to evaluate whether a patient has anemia. 5. Petechiae - these are small red or purple spots on the skin, usually associated with abnormal platelet count or coagulation issue. 6. Crackles at the right lung base - this is a sign of fluid accumulation in the lung, which could be due to several reasons, including infection or heart failure. 7. Cefuroxime, azithromycin, levofloxacin, ticarcillin, and piperacillin-tazobactam are all antibiotics with distinct bacterial coverage. PLANNING: Assumption 1 - The patient's symptoms are due to a single illness. Assumption 2 - That illness is infectious. Assumption 3 - Antibiotics are needed for treatment. Assumption 4 - There's a single best choice among the antibiotics listed. Questioning Forward: - Could her symptoms be due to more than one condition? - Could her symptoms be due to non-infectious causes? - Are antibiotics the best treatment option for her condition? - Could more than one antibiotic listed be appropriate for her condition? - Could the patient's recent travel or potential pregnancy affect our choice of therapy? CLINICAL REASONING: The patient's fever, cough, shortness of breath, and the presence of crackles suggest pneumonia. Pregnant women may be more susceptible to certain infections, though this patient has not confirmed her pregnancy with a diagnostic test other than the positive β-hCG. The lethargy, diarrhea, and elevated ALT/AST suggest a broader systemic infection or sepsis, not just an isolated pneumonia. With the presence of petechiae and decreased platelet count, there is potential that she might be developing disseminated intravascular coagulation (DIC), which often occurs in severe infections. All these symptoms might also suggest some form of atypical bacterial infection. Since there are findings of both liver and kidney involvement, we can hypothesize that this could be a systemic infection, possibly caused by leptospirosis, given her recent travel to a Midwestern city and the epidemiology of this bacteria in that region of the U.S. Leptospirosis is an infection caused by leptospira bacteria and influenza-like symptoms, liver and kidney disease, and rash could suggest this diagnosis. Therefore, antibiotic treatment suitable for addressing leptospirosis should be prioritized. CONCLUDING: Based on the clinical/laboratory findings and travel history, the clinical picture is suggestive of a more systemic infection potentially taxa and drug-resistance profiles in bacteria. We also need to consider the safety of the drug in pregnancy, ruling out any that might potentially harm the fetus. RESPONSE: Given these considerations, the most appropriate treatment would be (E) Piperacillin-tazobactam. It is an antibiotic that provides broad-spectrum coverage effective against many aerobic and anaerobic bacteria, including Leptospira, and relatively safe for use in pregnancy.

**Facilitator** **[Round 2]:** Summary: AI_A believes that the patient's symptoms suggest a respiratory infection, possibly community-acquired pneumonia (CAP), and a potential blood disorder or disseminated intravascular coagulation (DIC). Given the patient's likely pregnancy, AI_A recommends a combination of cefuroxime and azithromycin, which are generally safe in pregnancy and cover typical and atypical bacterial causes of pneumonia. AI_B also suggests that the patient's symptoms indicate community-acquired pneumonia and septicemia, as well as iron deficiency anemia. Like AI_A, AI_B recommends a combination of cefuroxime and azithromycin, which are effective against the pathogens usually involved in CAP and safe in pregnancy. AI_C, however, believes that the patient's symptoms suggest a severe lower respiratory tract infection that could have developed into sepsis. Given the severity of the symptoms and the potential for pregnancy, AI_C recommends piperacillin-tazobactam, which covers a broad range of organisms and is safe in pregnancy. AI_D suggests that the patient's symptoms indicate a systemic infection, possibly leptospirosis, given her recent travel to a Midwestern city. AI_D recommends piperacillin-tazobactam, which provides broad-spectrum coverage effective against many bacteria, including Leptospira, and is safe in pregnancy. AI_E believes that the patient's symptoms suggest pneumonia and a potential zoonotic disease, possibly histoplasmosis. Given the patient's likely pregnancy, AI_E recommends a combination of cefuroxime and azithromycin, which are effective against community-acquired pneumonia and safe in pregnancy. Key Differences: The key differences in the council members' opinions lie in their interpretation of the patient's symptoms and their recommended treatment. AI_A, AI_B, and AI_E recommend a combination of cefuroxime and azithromycin, while AI_C and AI_D recommend piperacillin-tazobactam. AI_C and AI_D believe that the patient's symptoms suggest a more severe or systemic infection, while AI_A, AI_B, and AI_E believe that the symptoms suggest pneumonia and possibly another condition. Question for Council Members: Given the differences in your interpretations of the patient's symptoms and your recommended treatments, could you explain why you believe your recommended treatment is the most effective and safe for the patient, considering her likely pregnancy and potential for a more severe or systemic infection?. Based on your preceding response, how would you answer the original question: [A 32-year-old woman comes to the emergency department because of a 3-day history of worsening fever, dry cough, and shortness of breath. She also has had abdominal discomfort, diarrhea, and nausea, but she has not vomited. She returned home from a business trip to a midwestern city 2 days ago. She has had no known contact with any ill individuals. She had been generally healthy and medical history is unremarkable. She takes no medications. Her last menstrual period was 6 weeks ago and she thinks she might be pregnant. She does not smoke cigarettes, drink alcoholic beverages, or use illicit drugs. She is married and uses no contraception. She appears well developed and well nourished but is short of breath and lethargic. Vital signs are temperature 38.3°C (101.0°F), pulse 104/min, respirations 28/min, and blood pressure 100/60 mm Hg. Pulse oximetry on room air shows an oxygen saturation of 92%. Auscultation of the chest discloses crackles at the right lung base, tachycardia, a normal S1 and S2, and no other abnormalities. Abdominal examination discloses no abnormalities. Petechiae are present over both lower extremities. Results of laboratory studies are shown: Serum ALT - 80 U/L AST - 63 U/L Urea nitrogen - 14 mg/dL Creatinine - 1.7 mg/dL Na⁺ - 130 mEq/L K⁺ - 3.9 mEq/L Cl⁻ - 104 mEq/L HCO3⁻ - 16 mEq/L Iron, total - 18 μg/dL Iron binding capacity, total - 428 μg/dL (N=250–350) Iron saturation - 5% (N=20–50) β-hCG - Positive Blood Hematocrit - 30.3% Hemoglobin - 10.2 g/dL WBC - 11,300/mm³ MCV - 76 μm³ Platelet count - 104,000/mm³ Red cell distribution width - 16% (N=11.5–14.5) Which of the following is the most appropriate intravenous pharmacotherapy at this time? (A) Cefuroxime alone (B) Cefuroxime and azithromycin (C) Levofloxacin alone (D) Levofloxacin and ticarcillin (E) Piperacillin-tazobactam]

**AI_A:** The question asks to identify the most suitable pharmacological therapy for our patient, a seemingly pregnant 32-year-old woman demonstrating symptoms characteristic of a respiratory infection, potentially community-acquired pneumonia, alongside abdominal discomfort, diarrhea, and nausea. She evidently also has petechiae, suggesting a possible disorder of the blood. The technical phrases in the question were largely centered around the patient's symptomatology and laboratory results, which reflected findings such as a positive β-hCG, indicative of likely pregnancy, pulmonary irregularities, along with an iron deficiency anemia and increased RDW, implying the existence of multiple pathologies. In examining possible assumptions, the first assumption regards the patient's symptomatology, which reflects a possible respiratory infection, likely community-acquired pneumonia (CAP). She also exhibits gastrointestinal discomfort, indicative of possible gastrointestinal involvement or systemic infection. The patient's petechiae suggest a possible blood disorder. Moreover, given the positive β-hCG test, the patient is likely pregnant. Each of these assumptions warrants consideration in the patient's treatment plan. In evaluating these assumptions, analyzing the severity of her symptoms alongside her pathology, it's evident this patient is likely dealing with a more critical condition, potentially sepsis or systemic infection that extends beyond pneumonia. This lends credence to the necessity of an effective and broad-spectrum pharmacotherapy that can manage potential pathogens involved. Therefore, having reasoned clinically that her condition is severe and her pregnancy status must be considered, treatment should encompass a broad-spectrum antibiotic safe in pregnancy. Given her potential sepsis status, antibiotic treatment should not only target CAP but consider her systemic symptoms and blood disorder as well. According to the above reasoning process, my response as AI_A is that the most appropriate intravenous pharmacotherapy for this patient at this moment would be (B) Cefuroxime and azithromycin. This therapy combination covers pathogens responsible for CAP, is safe in pregnancy, and addresses the patient's potential blood disorder. Can AI_B to AI_F further interrogate this assumption and provide their respective reasoning processes and final responses?

**AI_C:** To rephrase the question, we're being asked which intravenous medication should be administered immediately to a 32-year-old woman who might be pregnant and who is currently presenting a wide range of symptoms including fever, dry cough, breathing difficulties, abdominal discomfort, nausea, and diarrhea. The woman is also showing symptoms of anemia and coagulation irregularities. Moreover, she recently returned from a business trip from a midwestern city and has no history of contact with sick individuals. Key terms to understand in this scenario are: Cefuroxime, Azithromycin, Levofloxacin, Ticarcillin, and Piperacillin-Tazobactam – these are all antibiotics and are suggested as possible treatments. We can infer from the information available that the woman is showing broad symptoms that could indicate a severe infection potentially in both her respiratory and gastrointestinal systems. We can't overlook the fact that she is also likely pregnant. It is acknowledged that the pathology of her condition may be multifactorial. One possible assumption here is that her respiratory symptoms might be due to an atypical, probably community-acquired, pneumonia. Her gastrointestinal symptoms could indicate a gastrointestinal infection. The petechiae, thrombocytopenia, and her possible pregnancy status introduce complexities in clinical management and choice of antibiotics. An alternate assumption challenges that the symptoms may not be limited to pneumonia and gastrointestinal infection, they could be indicative of a systemic infectious process or a zoonotic infection, possibly acquired during her trip, which can additionally complicate the clinical picture. Assuming the presence of a possible systemic infection, with septicemia or zoonotic disease, and considering her pregnancy, careful selection of antibiotics that offer broad coverage, while being safe for use during pregnancy, should become a priority. Starting from the alternate assumption, we know her symptoms point towards a possible lower respiratory tract infection which could have triggered sepsis. Knowing that she recently traveled, the cause may well be a "travel-associated" disease, possibly a zoonotic infection, such as leptospirosis or histoplasmosis. Considering these clinical reasonings, it seems the most appropriate therapeutic course in this case would be to select an antibiotic or combination of antibiotics that offer coverage for possible lung and gastrointestinal pathogens, including atypical organisms, and is safe for a potentially pregnant woman. This therefore leads me to recommend the choice of combination therapy with Cefuroxime and Azithromycin (Option B). This combination will provide coverage against common pathogens of community-acquired pneumonia (including atypical pathogens covered by Azithromycin) and also the Enterobacteriaceae group which are often the cause of gastrointestinal infections. Both drugs are considered safe in pregnancy.

**AI_E:** Restating the question, we are examining the appropriate therapeutic response in a clinical scenario. The patient is a 32-year-old woman who is presenting with fever, dry cough, and shortness of breath along with abdominal discomfort, diarrhea, and nausea. She has not vomited and she has just returned from a business trip to a midwestern city. Moreover, her menstrual history suggests that she might be pregnant. Laboratory studies reveal that the patient is showing signs of preliminary lung disease and a blood disorder. We are asked to recommend the most suitable intravenous pharmacotherapy among the options provided which are cefuroxime alone, cefuroxime and azithromycin, levofloxacin alone, levofloxacin and ticarcillin, or piperacillin-tazobactam. Moving on to planning, the primary assumption is the woman's potential pregnancy and the possibility of her being inflicted with a form of lung disorder or blood disease. Both require different clinical prescriptions. Here, an alternate assumption could be the type of disease the patient is suffering from and its severity. It is this assumption, given the patient's conditions, that should be clinically prioritized. By clinical reasoning, we can start by assessing the likelihood of pregnancy which increases the risk of complications or contraindications with certain medications. Symptoms like fever, cough, and shortness of breath combined with detected anomalies in her lab results strongly suggest a respiratory infection. Besides, her travel history might indicate exposure to sources of infection common in the midwestern part of the country, potentially hinting at a zoonotic disease. The lab results also hint at a possible blood disorder, manifested by petechiae and the platelet count. As my conclusion, considering the symptoms and the potential for pregnancy, a suitable recommendation should cover both typical and atypical pneumonia pathogens but, at the same time, should prove relatively safe for the fetus. Among the listed choices, a combination of cefuroxime and azithromycin (option B) adequately meets these requirements. It is noteworthy that while piperacillin-tazobactam serves as a broad-spectrum antibiotic, it may be more prudent to initiate a conservative treatment approach with cefuroxime and azithromycin which sufficiently covers most causes of community-acquired pneumonia, since the patient's symptoms do not seem severe enough to necessitate broad-spectrum coverage from the get-go. So, my response would be: Given the patient's symptoms, travel history, and potential for pregnancy, the most appropriate intravenous pharmacotherapy at this time would be a combination of cefuroxime and azithromycin (option B).

**AI_D:** The presented scenario describes a patient, a probable pregnant 32-year-old woman, who is exhibiting symptoms such as worsening fever, dry cough, shortness of breath, abdominal discomfort, diarrhea, and nausea. There is no record of her having been in contact with any sick individuals, but she did recently return from a business trip. Some symptoms, along with certain lab results, indicate a possible infection. The blood studies highlight suspected anemia and low platelets, suggesting a possible coagulation issue. The question is which intravenous pharmacotherapy would be the most appropriate in her situation. Identifying antibiotic therapy assumes that this patient is suffering from a bacterial infection that must be treated urgently. Pneumonia could be suspected due to her respiratory symptoms and diminished oxygen saturation, along with the presence of crackles at her right lung base. Given her travel to a Midwest city, we need to consider possible exposure to diseases endemic in that region as well. The associated gastrointestinal symptoms, in conjunction with petechiae over the lower extremities, raise the possibilities of a systemic bacterial infection. Her pregnancy status and the safety of medications for the fetus should also be highly prioritized. Considering the systemic involvement and possible severe bacterial infection, broad-spectrum antibiotics such as piperacillin-tazobactam could be a first-line choice. This medication covers a wide variety of pathogens, including gram-positive, gram-negative, and anaerobic bacteria. Given her recent travel, leptospirosis could be considered, which is susceptible to penicillins like piperacillin. Also, piperacillin-tazobactam is considered safe in pregnancy (category B), thus not posing a high risk to the fetus. However, the alternate opinion would be to treat with respect to community-acquired pneumonia (CAP). CAP is typically caused by Streptococcus pneumoniae, Mycoplasma pneumoniae, or Haemophilus influenzae. Therefore, a combination of a beta-lactam such as cefuroxime (effective against Streptococcus pneumoniae and Haemophilus influenzae) and a macrolide like azithromycin (covers atypical bacteria like Mycoplasma pneumoniae) would typically be recommended. Both these drugs are categorized as pregnancy-safe category B. In conclusion, both piperacillin-tazobactam and the combination of cefuroxime and azithromycin seem to be reasonable choices based on her infection's suspected severity and etiology. Therefore, to answer the prompt, the most appropriate intravenous pharmacotherapy in this instance could be either (B) Cefuroxime and azithromycin if we are prioritizing treating CAP or (E) Piperacillin-tazobactam if we are considering a more severe or wide-spread infection. Given the current data, it might be hard to definitively conclude the best option, and either could be justifiable based on the clinical reasoning provided. However, considering that CAP is a common condition, we cannot overlook it, and therefore, the combination treatment of Cefuroxime and Azithromycin might be the more reasonable choice as it covers a wider range of CAP causative organisms.

**AI_B:** Restating the question: The query necessitates identifying the most suitable intravenous drug therapy option for a 32-year-old woman presenting symptoms of fever, dry cough, shortness of breath, abdominal discomfort, diarrhea, and nausea post a midwestern city business trip. No known contact history with sick individuals noted. She reveals that she might be pregnant as her last menstrual phase was six weeks ago paired with positive β-hCG results. She does not engage in smoking, alcohol consumption, or illegal drug usage. She is currently experiencing shortness of breath and lethargy. Her physical examination and laboratory results hint at a concerning infection, possibly pneumonia and a blood disorder. Her vital parameters and laboratory results, including slightly elevated AST and ALT, a little increased creatinine, decreased sodium and bicarbonate levels, signs of iron deficiency anemia, and low platelet count, all are significant. The question is to select the most suitable IV pharmacotherapy among Cefuroxime alone or combined with Azithromycin, Levofloxacin alone or combined with Ticarcillin, and Piperacillin-Tazobactam. Planning: The assumptions made based on the data is that the patient is experiencing pneumonia, potentially community-acquired (CAP), and underlying blood disorder, possibly disseminated intravascular coagulation (DIC) or iron deficiency anemia. The individual elements of the question suggest the possibility of a pulmonary infection secondary to a respiratory pathogen, compounded by a low iron status and DIC. This perspective would assume that the infection is either bacterial or viral. Further elucidation of the etiology of the infection could allow for a more targeted IV treatment. Clinical reasoning: To formulate an optimal patient-tailored clinical plan, we first need to understand the possible causes of the patient's symptoms. The symptoms reflect pneumonia and an underlying systemic illness that could be related to the patient's recent travel. Therefore, it will be important to analyze each assumption carefully, but based on the current data, any organism causing pneumonia could be responsible for the patient's illness, and it is likely bacterial. Pneumonia is a serious infection, particularly in pregnant women, and can lead to severe morbidity or even mortality if not appropriately treated. Given the patient's recent travel to a midwestern city, it is reasonable to consider a possible exposure to atypical pathogens, such as Mycoplasma pneumoniae or Legionella pneumophila. When we combine the respiratory symptoms and the laboratory findings suggestive of a systemic response (elevated WBC, slightly increased AST and ALT), the notion of a spreading bacterial infection (septicemia) comes into play. Conclusively, considering the differential diagnosis and possible contraindications for antibiotic use, especially during pregnancy, a thorough review of the effectiveness and safety of each treatment option is mandatory. Concluding: Upon reviewing the potential antibiotics, given the patient's potential pregnancy status and the severity of the symptoms, treatment requires effective coverage for common and atypical pathogens. Considering the common pathogens involved in CAP (Streptococcus pneumoniae, Haemophilus influenzae, Moraxella catarrhalis) and possible atypical ones (Mycoplasma pneumoniae, Chlamydia pneumoniae, Legionella pneumophila), a combination of Cefuroxime and Azithromycin appears to be a suitable choice. This combination provides coverage against these pathogens and is generally safer during pregnancy. While Levofloxacin could be a good choice for pneumonia, it's contraindicated in pregnancy due to potential cartilage damage in the fetus. Response: The most appropriate intravenous pharmacotherapy at this time is (B) Cefuroxime and Azithromycin. Given the patient's symptoms, the severity of the infection, and her potentially pregnant status, this combination provides a safe and effective treatment option for the probable community-acquired pneumonia.

**Facilitator** The consensus statement synthesizes the responses of AI_A to AI_E regarding the most suitable intravenous pharmacotherapy for a 32-year-old woman who is likely pregnant and presenting symptoms of a respiratory infection, potentially community-acquired pneumonia, along with abdominal discomfort, diarrhea, and nausea. She also has petechiae, suggesting a possible blood disorder. The patient's symptomatology and laboratory results, which reflect findings such as a positive β-hCG, indicative of likely pregnancy, pulmonary irregularities, along with an iron deficiency anemia and increased RDW, imply the existence of multiple pathologies. The primary assumption is that the patient's symptoms reflect a possible respiratory infection, likely community-acquired pneumonia (CAP), and a possible gastrointestinal involvement or systemic infection. The patient's petechiae suggest a possible blood disorder. Moreover, given the positive β-hCG test, the patient is likely pregnant. Each of these assumptions warrants consideration in the patient's treatment plan. The severity of her symptoms alongside her pathology suggests that this patient is likely dealing with a more critical condition, potentially sepsis or systemic infection that extends beyond pneumonia. This lends credence to the necessity of an effective and broad-spectrum pharmacotherapy that can manage potential pathogens involved. Given her potential sepsis status, antibiotic treatment should not only target CAP but consider her systemic symptoms and blood disorder as well. The treatment should encompass a broad-spectrum antibiotic safe in pregnancy. Considering the common pathogens involved in CAP (Streptococcus pneumoniae, Haemophilus influenzae, Moraxella catarrhalis) and possible atypical ones (Mycoplasma pneumoniae, Chlamydia pneumoniae, Legionella pneumophila), a combination of Cefuroxime and Azithromycin appears to be a suitable choice. This combination provides coverage against these pathogens and is generally safer during pregnancy. Therefore, the consensus among the AI responses is that the most appropriate intravenous pharmacotherapy for this patient at this moment would be (B) Cefuroxime and azithromycin. This therapy combination covers pathogens responsible for CAP, is safe in pregnancy, and addresses the patient's potential blood disorder.
